# TLR2 Expression in Peripheral CD4+ T Cells Promotes Th17 Response and Is Associated with Disease Aggravation of Hepatitis B Virus-Related Acute-On-Chronic Liver Failure

**DOI:** 10.3389/fimmu.2017.01609

**Published:** 2017-11-23

**Authors:** Chunli Xu, Yinping Lu, Xin Zheng, Xuemei Feng, Xuecheng Yang, Joerg Timm, Jun Wu, Baoju Wang, Mengji Lu, Dongliang Yang, Jia Liu

**Affiliations:** ^1^Department of Infectious Disease, Union Hospital, Tongji Medical College, Huazhong University of Science and Technology, Wuhan, China; ^2^Department of Anesthesiology, Union Hospital, Tongji Medical College, Huazhong University of Science and Technology, Wuhan, China; ^3^Institute for Virology, University Hospital, Heinrich-Heine-Universität Düsseldorf, Düsseldorf, Germany; ^4^Institute for Virology, University Hospital of Essen, University of Duisburg-Essen, Essen, Germany

**Keywords:** toll-like receptor 2, chronic hepatitis B, chronic hepatitis B-related liver failure, T helper cell 17, CD4+ T cells

## Abstract

Th17 responses have been shown to play crucial roles in the pathogenesis of hepatitis B virus (HBV)-associated acute-on-chronic liver failure (ACLF). The mechanism underlying the enhanced Th17 responses in these patients remains largely unclear. Here we investigated toll-like receptors (TLRs) expression in peripheral T cells and their roles in Th17 cell differentiation and disease aggravation in ACLF patients. 18 healthy subjects (HS), 20 chronic HBV-infected (CHB) patients, and 26 ACLF patients were enrolled and examined for TLRs expression in peripheral blood mononuclear cells (PBMCs). The correlations of T cell TLR2 expression with the antigen non-specific Th17 responses and disease aggravation, as well as the Th17 response to TLR2 ligand stimulation were evaluated in ACLF patients. Compared to HS and CHB patients, ACLF patients showed a distinct TLRs expression pattern in PBMCs. Significantly increased TLR2 expression in T cells was observed in ACLF patients. The TLR2 expression in CD4+ T cells was correlated with the Th17 responses and the clinical markers for disease aggravation in ACLF patients. Moreover, TLR2 ligands stimulation promoted Th17 cell differentiation and response in PBMCs of ACLF patients. These findings implicate that TLR2 signaling plays critical roles in Th17 cell differentiation and disease aggravation of HBV-related ACLF.

## Introduction

It is estimated that more than 248 million people are chronically infected with Hepatitis B virus (HBV) worldwide (1). Chronic HBV infection leads to severe sequelae including chronic liver failure, acute-on-chronic liver failure (ACLF), liver cirrhosis, and hepatocellular carcinoma (2, 3). Among them, ACLF is characterized by acute onset, poor prognosis and high short-term mortality, ranging from 50 to 90% (4, 5). It is defined as an acute liver decomposition with jaundice, coagulation dysfunction, hepatic encephalopathy, and/or ascites within 4 weeks on the basis of chronic liver disease (5). Clarifying the mechanism of HBV-related ACLF is very important for the treatment and prevention of the disease, and hence possesses great value.

So far, the exact mechanism of the development of HBV-related ACLF is still unclear. It is generally believed that the liver injury and the disease progression are intensively associated with the disordered inflammatory responses (6, 7). Current hypotheses suggest that the dysfunctional state of immune system in ACLF is very similar to that of severe sepsis, which is a dynamic and paradoxical coexistence of not only an exaggerated inflammatory response but also an immune paralysis (7, 8). T helper 17 (Th17) cells have been identified and characterized as a distinct CD4+ T cell lineage mediating tissue inflammation for more than 10 years (9, 10). Th17 produce a large amount of inflammatory cytokines, such as interleukin (IL)-17, IL-21, IL-22, and TNF-α, which induce the recruitment of immune cells to sites of inflammation and result in inflammatory injury (11, 12). Growing evidences suggest that Th17 cells are involved in the pathogenesis of HBV-related ACLF. For example, serum IL-17 concentration and Th17 cells frequencies in peripheral blood and liver were significantly upregulated and positively correlated with the severity of liver injury in HBV-related ACLF patients (13–17). However, little is known on how the Th17 response in these patients is regulated.

Toll-like receptors (TLRs) are a group of evolutionary conserved receptors that play a crucial role in the recognition of pathogen-associated molecular patterns and mediate the innate immune responses against invading infectious agents (18). Expression of TLRs on immune cells are related to the uptake and processing the different endogenous and exogenous antigens. Upon binding to specific ligands, TLRs trigger the activation of complex networks of intracellular signal transduction pathways to coordinate the ensuing inflammatory responses (19). Recently, TLRs have been found to induce IL-17 and IL-22 production by γδ T cells (20). Furthermore, TLR signaling in CD4+ T cells promotes Th17 responses and regulates the pathogenesis of disease in the experimental autoimmune encephalomyelitis model (21). Correspondingly, accumulating evidences demonstrated that altered TLRs expression on peripheral blood mononuclear cells (PBMCs) in ACLF patients (22–24). These reports suggest that TLRs may play an important role in promoting Th17 response in HBV-related ACLF patients and thus regulate the disease development and aggravation.

In the current study, we compared circulating Th17 cell frequencies and TLRs expression of CD4 and CD8 T cells in healthy subjects (HS), chronic HBV-infected (CHB) patients, and ACLF patients. We also evaluated the correlation of TLR2 expression on T cells and the disease aggravation in ACLF patients. Finally, we investigated whether TLR2 agonist stimulation promoted Th17 response in ACLF patients.

## Materials and Methods

### Subjects

46 HBV-infected patients, including 20 CHB patients and 26 ACLF patients were recruited at the in- and out-patient clinic of Department of Infectious Disease, Union Hospital, Tongji Medical College, Huazhong University of Science and Technology from May 2013 to March 2015 for this single-center study. The CHB patients were diagnosed as patients with previous history of clinical course of HBV infection or HBV surface antigen (HBsAg) positive for over 6 months, and currently remains positive for HBsAg and/or HBV DNA (25). The diagnosis of HBV-related ACLF patients was based on the APASL criteria, which is a history of CHB with acute hepatic insult manifesting as jaundice (serum bilirubin ≥ 85 μmol/L) and coagulopathy [international normalized ratio (INR) ≥ 1.5 or prothrombin activity <40%], complicated within 4 weeks by ascites and/or encephalopathy (26). These patients also met the newly proposed diagnostic criteria specific for HBV-related ACLF based on a prospective study performed in a large cohort of patients (1,322 cases) (27). Only patients without antiviral or immunotherapy treatment 6 months prior to sampling were included in the study. All patients were tested negative for human immunodeficiency virus, hepatitis C virus, hepatitis E virus, and hepatitis delta virus. Patients with alcoholic liver disease, autoimmune disease, malignancy, or serious illness of other systems were excluded. All patients received integrative treatment after hospital admission, including nucleoside analogs for HBV DNA-positive patients; a high-calorie diet; sodium restriction, diuretics, and paracentesis combined with albumin infusion for ascites; lactulose and l-ornithine aspartate for HE and prophylactic antibiotics for bacterial infections. Informed consent was obtained from each patient, and the study protocol was approved by the local medical ethics committee of Tongji Medical College, Huazhong University of Science and Technology in accordance with the guidelines of the Declaration of Helsinki (2015LSZ-022). For all ACLF patients, the blood samples were collected once within 24 h after hospital admission. Among them, nine patients were collected for blood samples every 7 days till hospital discharge or the patient death. The detailed clinical parameters of each ACLF patient were provided in Table S1 in Supplementary Material. Eighteen age- and gender-matched HS were enrolled as controls. PBMCs of HS and patients were isolated using Ficoll density gradient centrifugation (DAKEW Biotech, Beijing).

### RNA Extraction and Real-Time Reverse-Transcriptase Polymerase Chain Reaction (RT-PCR)

Total RNA was isolated using Trizol RNA reagent (Takara Bio Inc., Tokyo, Japan) according to the manufacturers’ protocol (28). One-step RT-PCR was carried out with the SYBR green real-time RT-PCR master mix (Toyobo, Osaka, Japan). The PCR primers of TLR1-10 and GAPDH were purchased from commercial Quantitec primer assays (Qiagen, Hilden, Germany).

### Cell Surface and Intracellular Staining by Flow Cytometry

Surface and intracellular staining were performed as described previously (24, 29). For TLR2 ligand stimulation, 1 × 10^6^ PBMCs were stimulated for 5 h with 50 ng/mL phorbol 12-myristate 13-acetate (PMA, Sigma, St. Louis, MO, USA), 1 µg/mL ionomycin (Sigma-Aldrich) and 10 µg/mL Brefeldin A (eBioscicence) in 200uL RPMI 1640 medium supplemented with 10% FCS. 1 µg/mL TLR2 ligand Pam3Cys was added to the stimulation media or not as indicated.

### Determination of Serum Cytokine Concentrations

Serum concentrations of the cytokines IFN-α, IL-10, TGF-β, and interleukin-6 (IL-6) were examined by commercial ELISA kits (Dakewe Bioengineering, Wuhan, China) according to the manufacturers’ protocols.

### Serological Assays and HBV DNA Assays

The presence of HBsAg, HBeAg, and anti-HBe was determined using commercial AxSYM MEI kits (Abbott Laboratories, Shanghai, China). HBV DNA was measured using the Roche Diagnostics COBAS TaqMan 48 (Meylan, France).

### Statistical Analysis

All data were analyzed using SPSS software 17.0 (SPSS Inc., Chicago, IL, USA). Multiple comparisons were made between the different groups using Mann–Whitney *U*-test, whereas comparisons between the same individual were made using the Wilcoxon matched pairs *t*-test. Categorical variable was conducted using chi-square tests. Spearman’s correlation test was used to assess the correlation of immune factors and clinical characters.

## Results

### Distinct TLRs Expression Pattern of PBMCs in HBV-Related ALCF Patients

**Figure 1:**
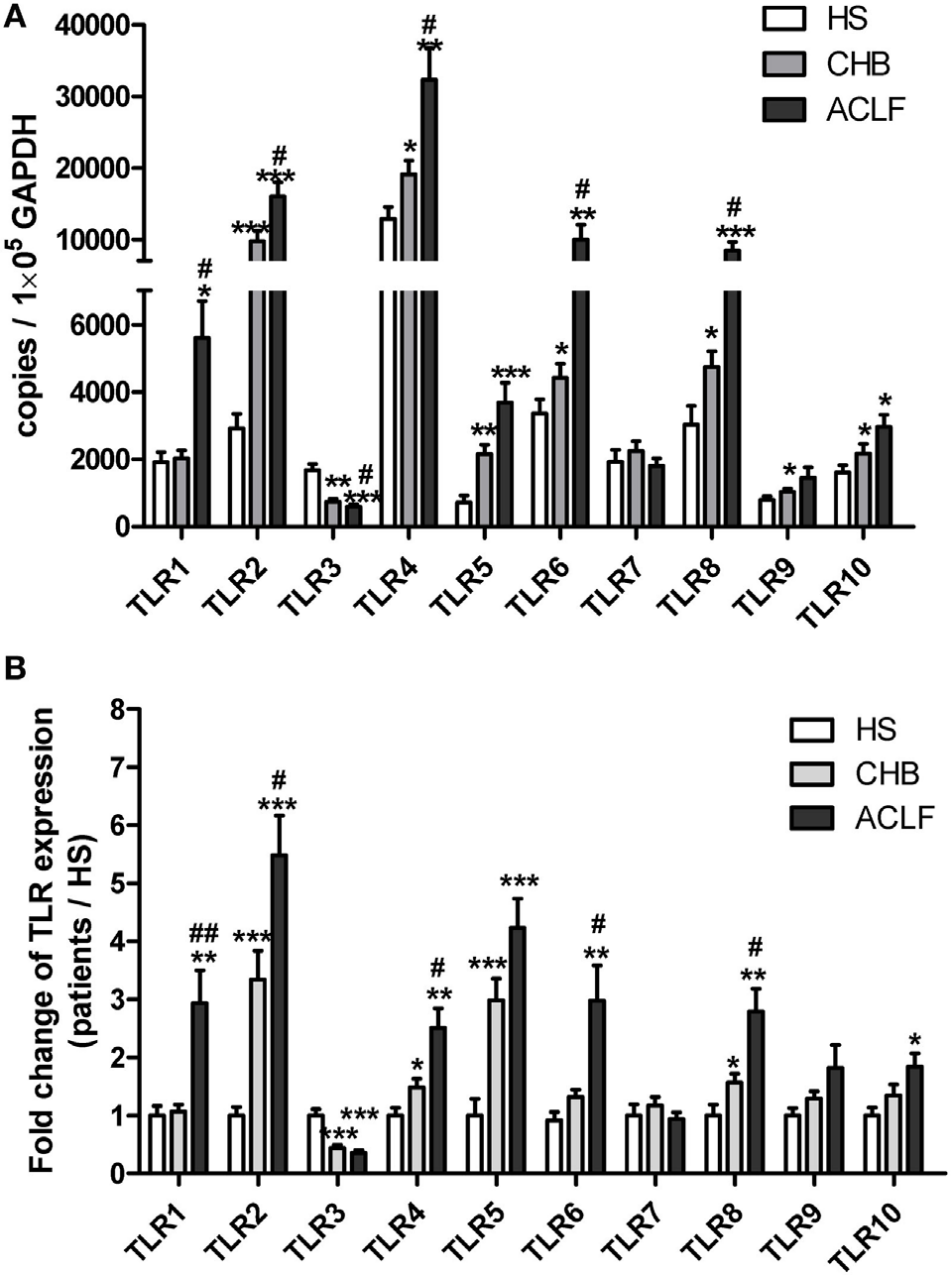
Distinct TLR1–10 expression pattern of peripheral blood mononuclear cells (PBMCs) in hepatitis B virus (HBV)-related ALCF patients. The TLR1–10 mRNA levels of PBMCs in 18 HS, 20 chronic HBV-infected (CHB) patients, and 26 HBV-related acute-on-chronic liver failure (ACLF) patients were determined by real-time reverse-transcriptase polymerase chain reaction (RT-PCR). **(A)** The absolute copy numbers of TLR1–10 transcripts per 100,000 GAPDH were calculated. **(B)** Fold changes of TLR1–10 expression of CHB and ACLF patients compared to HS were calculated. The TLR1–10 expression levels of HS were set to 1. Statistics analysis was performed by Mann–Whitney *U*-test. *P < 0.05, **P < 0.01, ***P < 0.001, compared to HS group; ^#^*P* < 0.05, ^##^*P* < 0.01, compared to CHB group. Only differences with statistical significances were marked.

To characterize the TLR expression pattern in HBV-related ACLF patients, a total of 18 HS, 20 CHB patients, and 26 HBV-related ACLF patients were recruited in this study (Table 1). The TLR1-10 mRNA levels of PBMCs in different groups of subjects were determined by real-time RT-PCR. In comparison with HS, CHB patients showed an altered TLRs expression pattern with increased TLR2/4/5/6/8/9/10 levels and decreased TLR3 level (Figure 1A). In HBV-related ACLF patients, further significant increase of TLR2/4/6/8 expression and decrease of TLR3 expression were observed compared to CHB patients. TLR5/10 expression in ACLF patients was significantly increased compared to HS but not to CHB patients. Although higher TLR9 expression was observed in ALCF patients compared to that of HS and CHB patients, the differences were not statistically significant. Besides, significantly higher TLR1 expression was observed only in ACLF patients than that of CHB patients and HS (Figure 1A). Among all TLRs, TLR2 was increased at the highest level in ACLF patients with a 5.5-fold increase compared to HS (Figure 1B). This result indicated that a distinct TLRs expression pattern of PBMCs with increased TLR1/2/4/5/6/8/10 and decreased TLR3 levels is present in HBV-related ACLF patients.

**Table 1:** Baseline characteristics of the study subjects.

Parameters	HS (*n* = 18)	CHB (*n* = 20)	ACLF (*n* = 26)	*P* value
Age, years	30 (25–50)	40 (18–58)	43 (34–66)	NS^α^
Gender, male/female	15/3	18/2	24/2	NS^α^
ALT, IU/L	17 (15–38)	60 (16–520)	102 (53–2,156)	<0.011
T-Bil, μmol/L	11 (6–18)	36.25 (15.2–84.5)	452.2 (218.5–859.7)	<0.0001
PT (s)	NA	13.4 (10.5–16.8)	24.2 (20.5–51)	<0.0001
PTA (%)	NA	78 (45–100)	25 (12–40)	<0.0001
INR, NO (%)	NA	1.04 (0.99–1.42)	2.15 (1.87–2.59)	<0.0001
HBeAg (+/−)	NA	10/10	10/16	NS^α^
HBV DNA, IU/ml	NA	5.6E + 05 (<5.0E + 02–5.8E + 06)	6.3E + 06 (4.0E + 04–4.7E + 08)	NS
Encephalopathy (%)	NA	0	50%	<0.0001^α^
Ascites (%)	NA	0	80.8%	0.001^α^
28-day mortality (%)	NA	0	19.2%	<0.0001^α^

*ACLF, acute-on-chronic hepatitis B; ALT, alanine aminotransferase; TBIL, total bilirubin; INR, International normalized ratio; PTA, prothrombin time activity; PT, prothrombin time; NA, not available; CHB, chronic HBV-infected; HBV, hepatitis B virus*.

*Data are shown as median and range. Statistics analysis was performed by Mann–Whitney U-test or chi-square test^α^ as indicated*.

### Increased TLR2 Expression on T Cells in HBV-Related ALCF Patients

**Figure 2:**
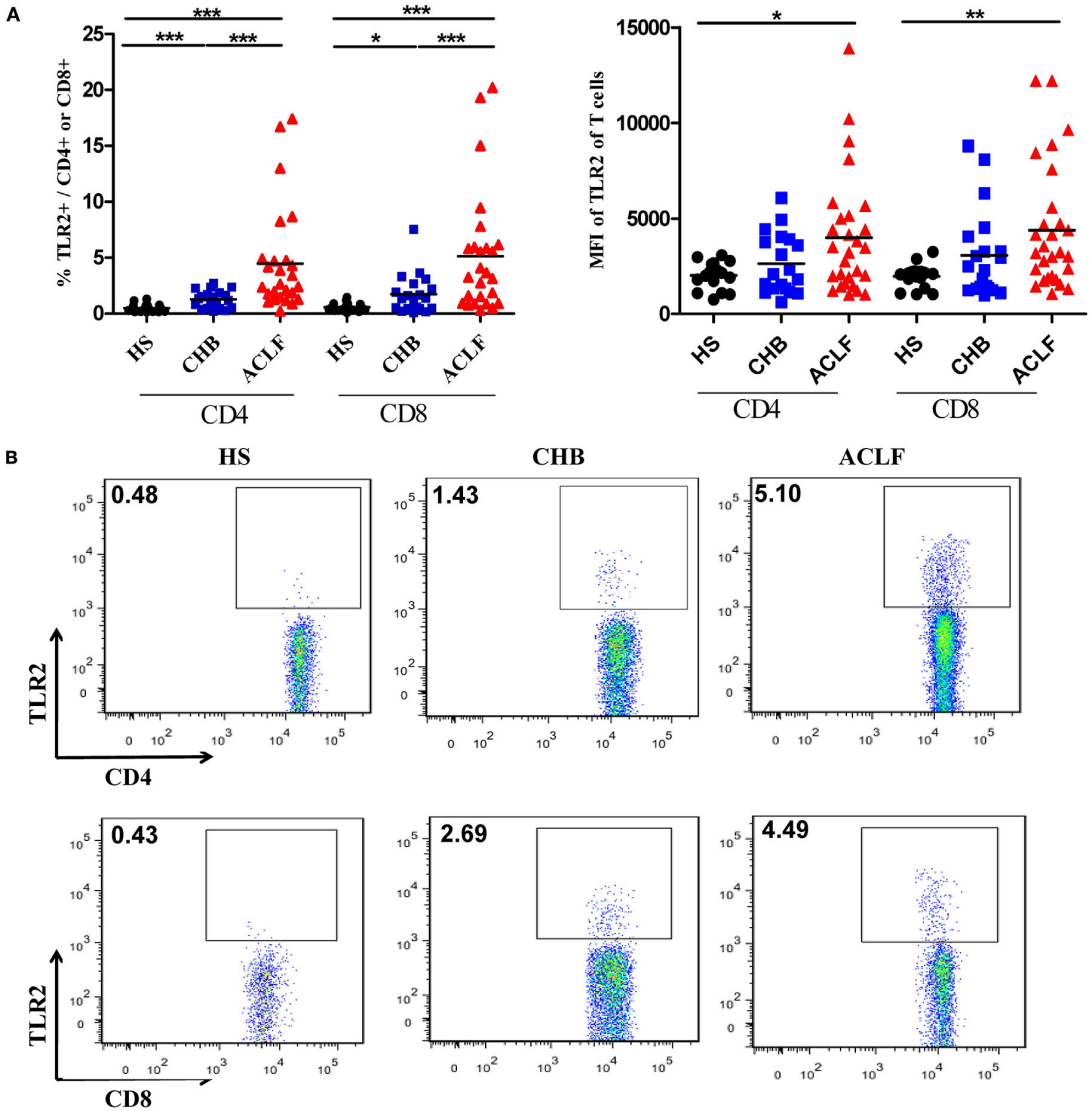
TLR2 expression on CD4+ T cells and CD8+ T cells of peripheral blood mononuclear cells (PBMCs) in the HS, chronic HBV-infected (CHB) patients and acute-on-chronic liver failure (ACLF) patients. The TLR2 expression on T cells of PBMCs in the HS (black dots), CHB patients (blue squares) and ACLF patients (red triangles) was determined by flow cytometry. The frequencies and the mean fluorescence intensity **(A)**, and the representative dot plots **(B)** of TLR2 expression on CD4+ T cells and CD8+ T cells are shown. *P < 0.05, **P < 0.001, ***P < 0.0001. Only differences with statistical significances were marked. Statistics analysis was performed by Kruskal–Wallis test and Mann–Whitney *U*-test.

**Figure 3:**
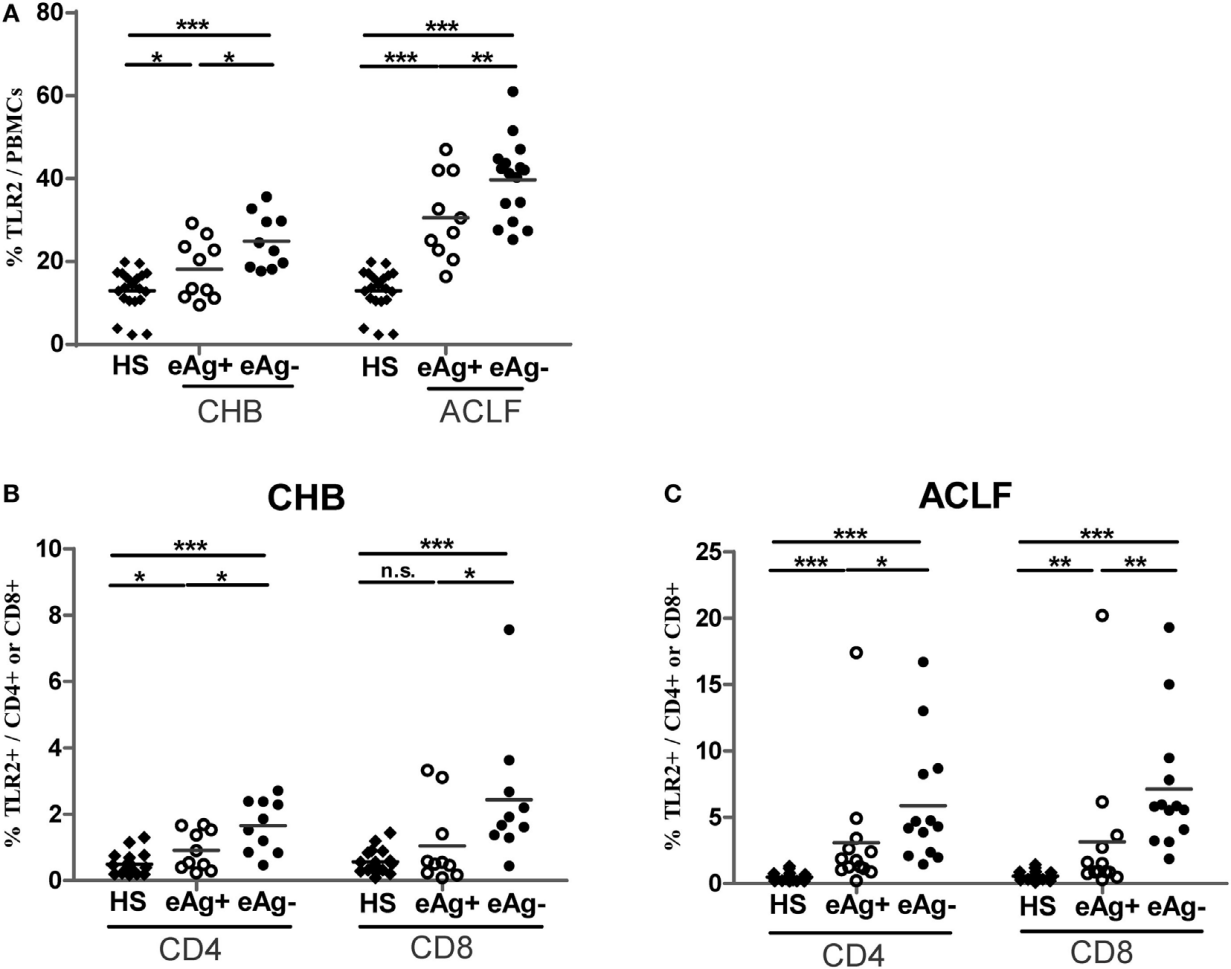
Comparison of TLR2 expression of peripheral blood mononuclear cells (PBMCs) and T cells between HBeAg+ and HBeAg− patients. The frequencies of TLR2 expression on PBMCs **(A)**, CD4+ T cells and CD8+ T cells in the HBeAg+ and HBeAg− patients of chronic HBV-infected (CHB) **(B)** and acute-on-chronic liver failure (ACLF) **(C)** groups were determined by flow cytometry. *P < 0.05, **P < 0.01, ***P < 0.001. Statistics analysis was performed by Mann–Whitney *U*-test.

Next, we examined TLR2 expression in various subsets of PBMCs from CHB and ACLF patients by flow cytometry. Consistent with previous report (30), we observed that the most frequent TLR2+ cells in PBMCs are CD14+ monocytes (data not shown). Although the frequency was significantly higher (Figure S1A in Supplementary Material), the mean fluorescence intensity (MFI) of TLR2 expression on monocytes of the ACLF patients was significantly lower than that of the HS. This means that the average TLR2 expression on single monocyte of ALCF patients was less than that of HS (Figure S1B in Supplementary Material). In contrast, there was a significant increase in TLR2 expression on both CD4+ and CD8+ T cells by frequency and MFI in ACLF patients than that in the HS (Figures 2A,B). ACLF patients also showed higher TLR2 expression on T cells by frequency than CHB patients (Figures 2A,B). The frequency of TLR2 expression on T cells in CHB patients was slightly higher than that in the HS (Figures 2A,B). It was previously reported that TLR2 expression in chronic HBV infection was regulated by HBeAg (31). Therefore, we next evaluated the impact of the presence of HBeAg on TLR2 expression in PBMCs and T cells in CHB and ACLF patients. We observed that both HBeAg-negative CHB patients and ACLF patients showed significant upregulation of TLR2 in total PBMCs compared with those of HBeAg-positive CHB patients and ACLF patients, respectively (Figure 3A). Both CD4+ and CD8+ T cells of HBeAg-negative CHB patients demonstrated significant upregulation of TLR2 compared with those of the HS controls and HBeAg-positive CHB patients (Figure 3B). In ACLF patients, both HBeAg-positive and HBeAg-negative groups showed significantly increased TLR2 expression on CD4+ and CD8+ T cells than HS. However, significantly higher TLR2 expression on CD4+ and CD8+ T cells of HBeAg-negative patients than that of HBeAg-positive patients was observed (Figure 3C). Taken together, our results indicated that HBeAg might negatively regulate TLR2 expression on T cells in both CHB and ACLF patients.

### Increased Th17 Frequency Is Positively Correlated with Liver Injury in HBV-Related ACLF Patients

**Figure 4:**
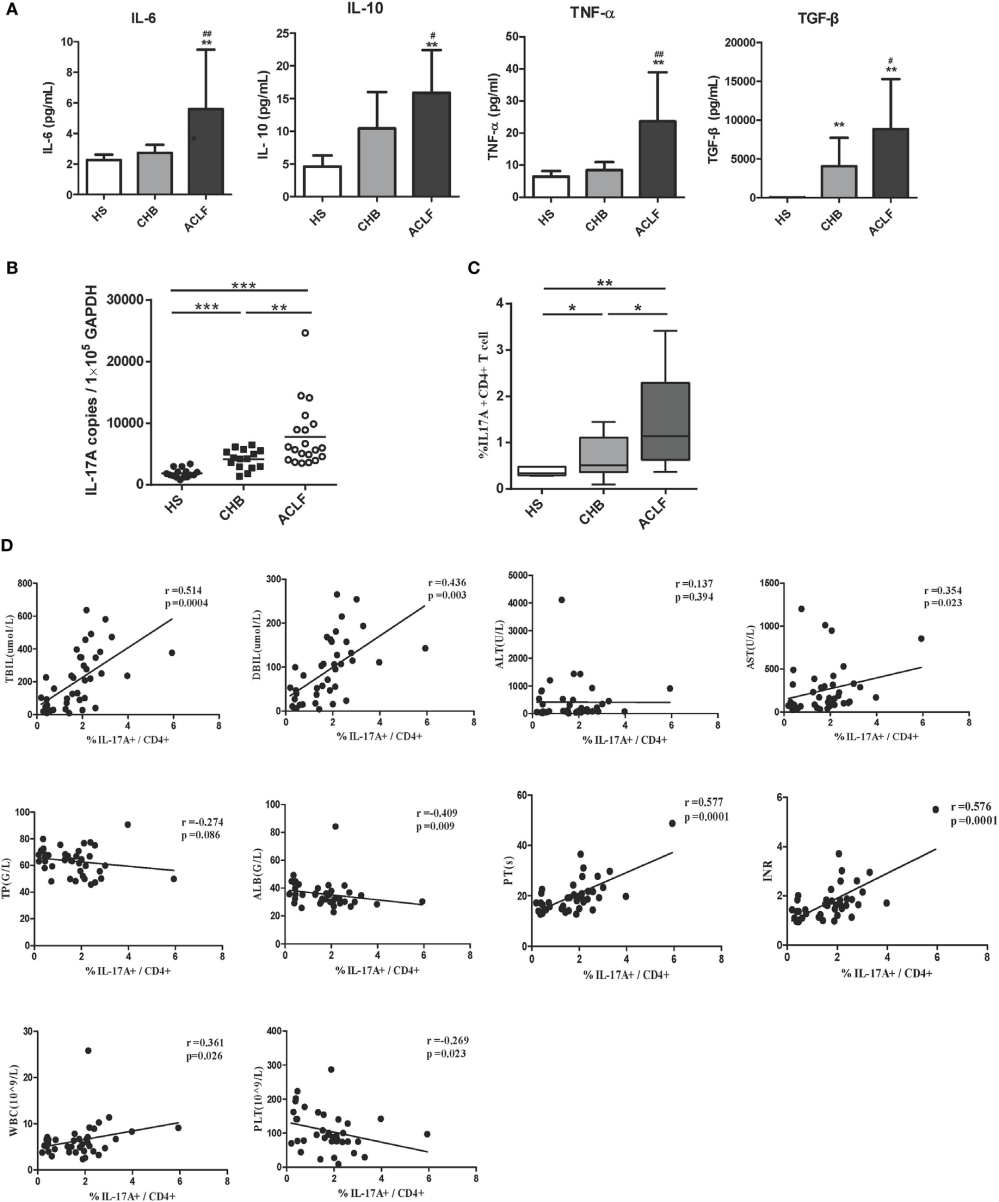
Th17 response in hepatitis B virus (HBV)-related acute-on-chronic liver failure (ACLF) patients. **(A)** ELISA were performed to quantify plasma interleukin-6 (IL-6), TGF-β, TNF-α, and IL-10 levels in HS (*n* = 18), chronic HBV-infected (CHB) patients (*n* = 20), and ACLF (*n* = 26) patients. **(B)** The IL-17A mRNA levels of peripheral blood mononuclear cells (PBMCs) in HS, CHB, and ACLF patients were determined by real-time reverse-transcriptase polymerase chain reaction (RT-PCR). **(C)** The frequencies of Th17 cells (IL-17+ CD4+ T) of PBMCs in HS, CHB, and ACLF patients were determined by flow cytometry. **(D)** The correlation analysis between peripheral Th17 cell frequencies and clinical parameters including TBIL, direct bilirubin (DBIL), ALT, AST, total protein (TP), serum albumin (ALB), prothrombin time (PT), international normalized ratio (INR), white blood cell (WBC), and platelet (PLT) was performed in all HBV infection patients. Solid line, linear growth trend; R, correlation coefficient. *P*-values are shown. *P < 0.05, **P < 0.01, ***P < 0.001, compared to HS group; ^#^*P* < 0.05, ^##^*P* < 0.01, compared to CHB group. Statistics analysis was performed by Mann–Whitney *U*-test. Pearson correlation test was used for correlation analysis.

Previous studies documented that the peripheral Th17 cell frequency and response in PBMCs were markedly increased in ACLF and CHB patients, and exhibit a potential to exacerbate liver damage (15, 17). We therefore examined the peripheral Th17 cell frequency and response in our investigation subjects. First, higher levels of IL-6, transforming growth factor-beta (TGF-β), and tumor necrosis factor-α (TNF-α), which are primarily related to Th17 cell differentiation, were observed in the sera of HBV-related ACLF patients than those of HS and CHB patients (Figure 4A). In consistent with these findings and the results of previous studies, significantly increased IL-17 mRNA levels and Th17 cell frequency were observed in HBV-related ACLF patients than that in HS and CHB patients (Figures 4B,C). Moreover, the increased Th17 frequencies were positively correlated with the total bilirubin (TBIL) levels (*r* = 0.514, *P* = 0.004), direct bilirubin (DBIL) levels (*r* = 0.436, *P* = 0.003), AST levels (*r* = 0.354, *P* = 0.023), prothrombin time (PT) (*r* = 0.577, *P* = 0.0001), INR (*r* = 0.576, *P* = 0.0001), and white blood cell (WBC) levels (*r* = 0.361, *P* = 0.026), and were negatively correlated with the serum albumin (ALB) levels (*r* = −0.409, *P* = 0.009) and platelet (PLT) levels (*r* = −0.269, *P* = 0.023) in all HBV-infected patients (Figure 4D). Thus, these data confirmed the positive correlation of Th17 cell response with liver injury in our investigation subjects.

### TLR2 Expression Levels on T Cells of PBMCs Is Positively Correlated with Th17 Response and Liver Injury in HBV-Infected Patients

**Figure 5:**
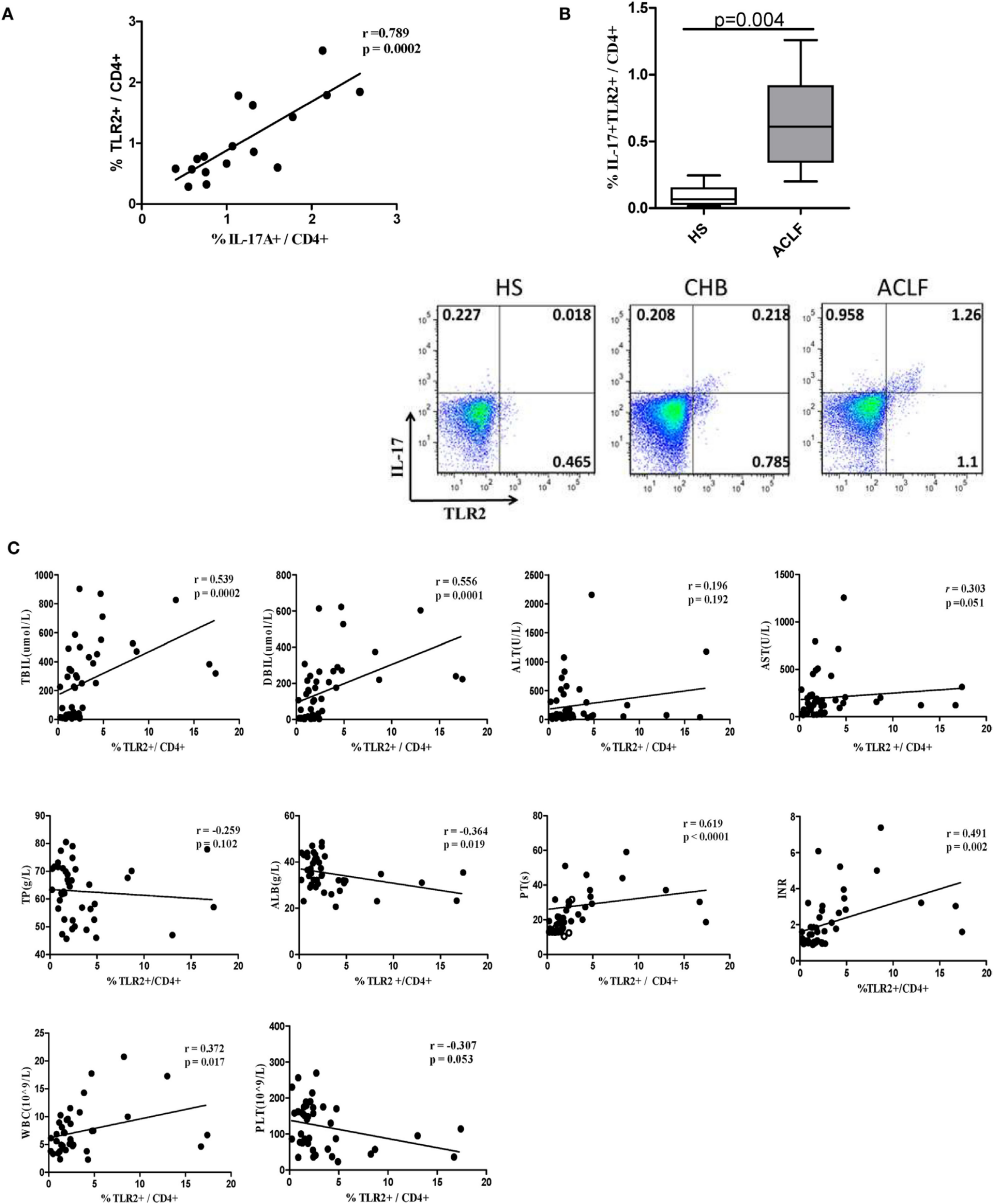

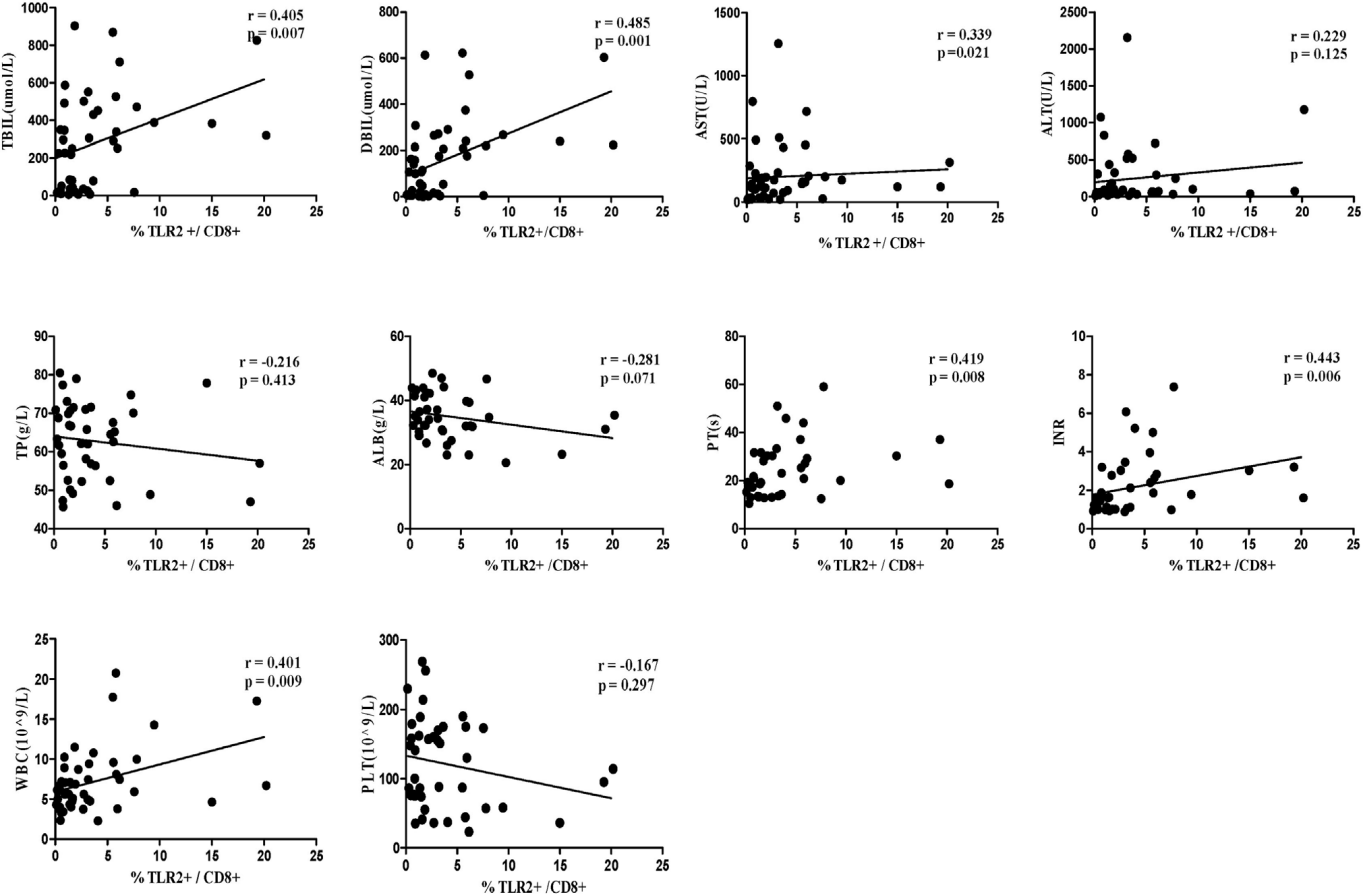
TLR2 expression levels on T cells of peripheral blood mononuclear cells (PBMCs) is positively correlated with Th17 response and liver injury in hepatitis B virus (HBV)-infected patients. **(A)** The correlation analysis between the frequencies of Th17 cells and TLR2+ CD4+ T cells was performed in the acute-on-chronic liver failure (ACLF) patients. **(B)** The frequency of IL-17A and TLR2 double positive CD4 T cells was analyzed by flow cytometry. **(C)** The correlation analysis between the frequencies of TLR2+ T cells and clinical parameters including TBIL, direct bilirubin (DBIL), ALT, AST, total protein (TP), serum albumin (ALB), prothrombin time (PT), international normalized ratio (INR), white blood cell (WBC), and platelet (PLT) was performed in all HBV infection patients. Solid line, linear growth trend; R, correlation coefficient. *P*-values are shown. Statistics analysis was performed by Mann–Whitney *U*-test. Pearson correlation test was used for correlation analysis.

It has been shown that TLR2 signaling in CD4+ T lymphocytes promotes Th17 responses in an autoimmune disease mouse model (21). Accordingly, we hypothesized that elevated Th17 response and liver injury might be associated with increased TLR2 expression on CD4+ T cells in HBV-related ACLF patients. We found that the percentage of TLR2+ CD4+ T cells was positively correlated with the frequency of Th17 cells in the ACLF patients (*r* = 0.789, *P* = 0.0002) (Figure 5A). Interestingly, significantly higher percentage of TLR2+ IL17+ CD4 T cells in ACLF patients than that in the HS was also observed (Figure 5B), indicating that the increased TLR2 expression on CD4+ T cells in ACLF patients may facilitate the Th17 cell differentiation. Moreover, the serum TBIL, DBIL, ALT, AST, PT, INR, PLT, WBC, total protein (TP), and ALB levels were measured to assess the liver injury severity and liver function of the CHB and ACLF patients, and were examined for their correlation with TLR2 expression on T cells. The result showed that TLR2 expression on both CD4+ and CD8+ T cells was positively correlated with serum TBIL (CD4: *r* = 0.539, *P* = 0.0002; CD8: *r* = 0.405, *P* = 0.007), DBIL (CD4: *r* = 0.556, *P* = 0.0001; CD8: *r* = 0.485, *P* = 0.001), AST (CD4: *r* = 0.303, *P* = 0.051; CD8: *r* = 0.339, *P* = 0.021), PT (CD4: *r* = 0.619, *P* < 0.0001; CD8: *r* = 0.419, *P* = 0.008), INR (CD4: *r* = 0.491, *P* = 0.002; CD8: *r* = 0.443, *P* = 0.006), and WBC (CD4: *r* = 0.372, *P* = 0.017; CD8: *r* = 0.401, *P* = 0.009) levels. TLR2 expression on CD4+ T cells but not CD8+ T cells was negatively correlated with ALB (*r* = −0.364, *P* = 0.019) and PLT (*r* = −0.307, *P* = 0.05) levels. No correlation was observed between TLR2 expression on T cells with serum ALT and TP levels (Figure 5C). These data indicate that increased levels of TLR2 expression on T cells might contribute to the liver injury and disease aggravation in HBV-related ACLF patients.

**Figure 6:**
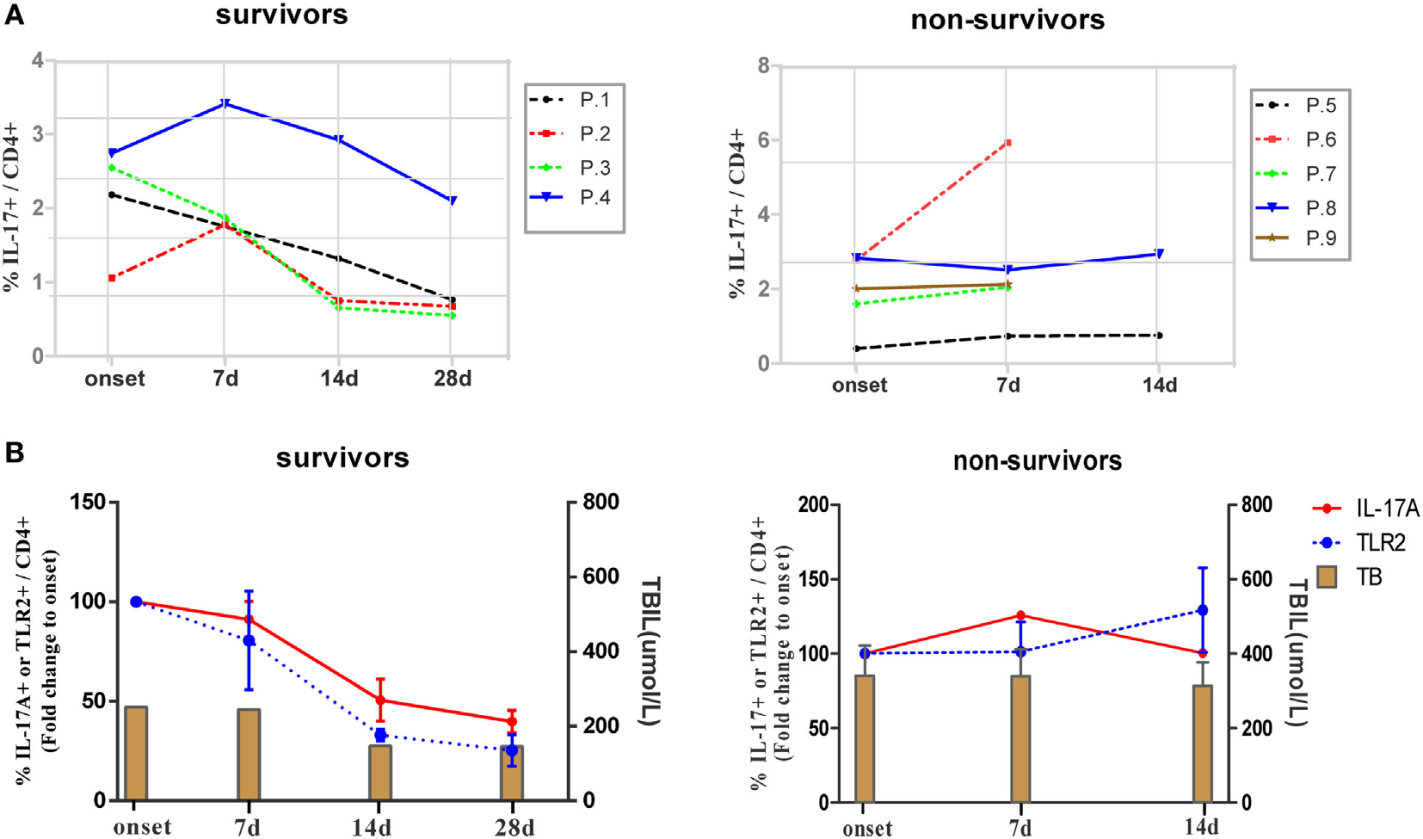
Kinetics of IL-17A and TLR2 expression on CD4+ T cells during hepatitis B virus (HBV)-related acute-on-chronic liver failure (ACLF). **(A)** Kinetics of Th17 cell frequencies in ACLF patients with different disease outcomes. **(B)** Kinetics of fold changes of TLR2+ CD4+ T cell frequency and Th17 cell frequency together with serum TBIL levels in ACLF patients with different disease outcomes are shown. The frequencies of TLR2+ CD4+ T cells and Th17 cells of disease onset were set to 100%.

Next, we longitudinally examined the antigen non-specific Th17 responses and TLR2 expression levels of CD4+ T cells in ACLF patients with different disease outcomes. Nine ACLF patients, including four survivors and five non-survivors, were monitored for Th17 responses and percentages of TLR2+ CD4+ T cells in PBMCs from the onset of the disease up to 28 days after. As shown in Figure 6A, four survivors with relieved disease aggravation showed gradually declined Th17 responses since either the onset of the disease (P.1 and P.3) or 7 days after (P.2 and P.4). In contrast, no drop of Th17 response was observed in all non-survivors. The kinetics of Th17 responses also coincided with the disease aggravation as indicated by serum TBIL levels in the two groups of patients (Figure 6B). We then examined the kinetics of TLR2 expression on CD4+ T cells in these patients, and not surprisingly, a continuous decline of TLR2 expression on CD4+ T cells was observed along with the decreasing Th17 responses in the survivors. In comparison to the average level of the onset point of the disease, the frequency of TLR2 expressing CD4+ T cells dropped 20% on day 7, 67% on day 14, and 75% on day 28 in survivors. In contrast, deteriorated patients showed maintained TLR2 expression of CD4+ T cells on day 7 and 30% increase on 14 days (Figure 6B). Collectively, our data indicated that the TLR2 expression levels on T cells of PBMCs are positively correlated with Th17 responses and disease aggravation in ACLF patients.

### TLR2 Signaling Enhances Th17 Response in HBV-Related ACLF Patients

**Figure 7:**
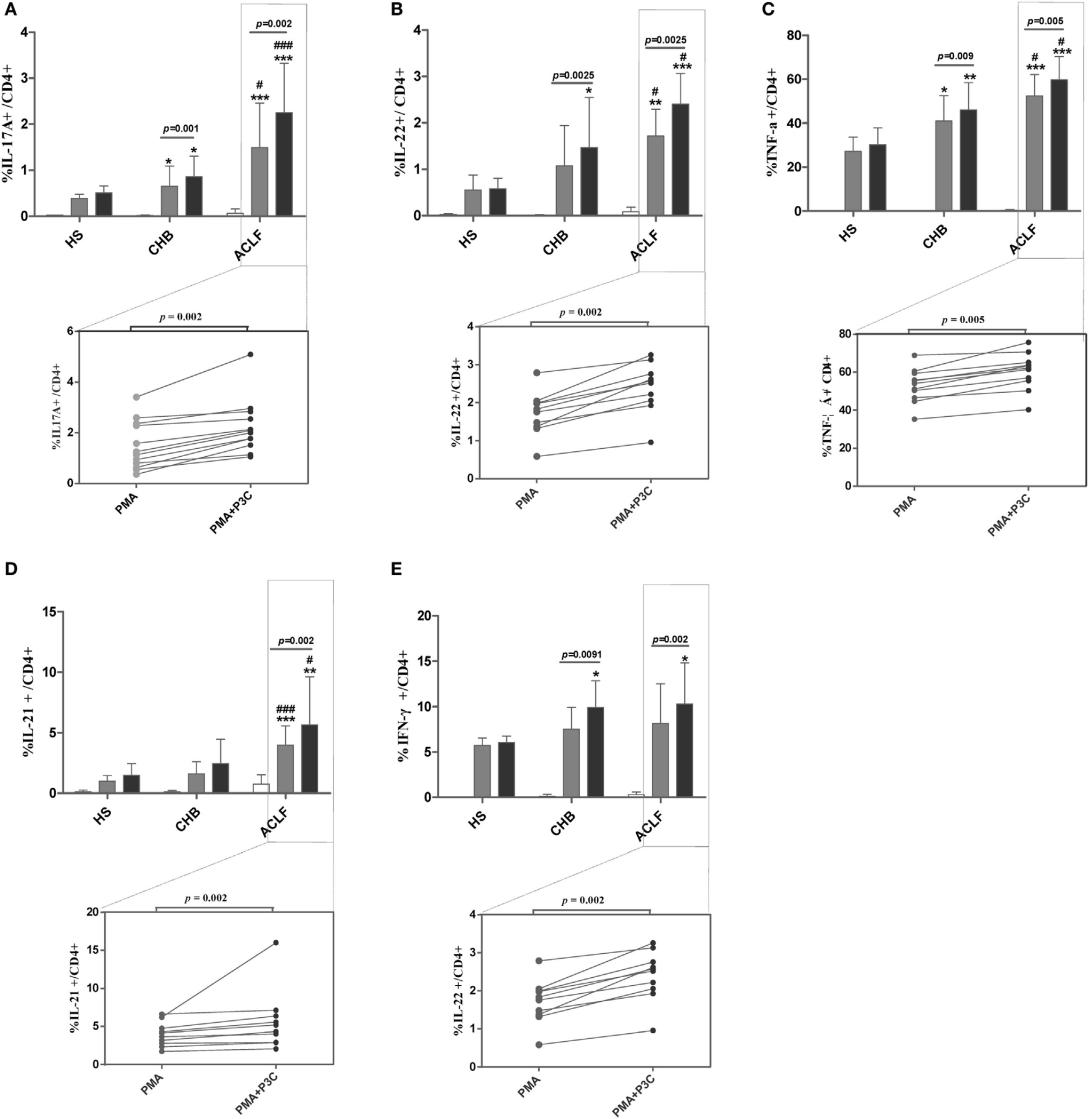
TLR2 signaling enhances Th17 response in hepatitis B virus (HBV)-related acute-on-chronic liver failure (ACLF) patients. Peripheral blood mononuclear cells (PBMCs) isolated from HS (*n* = 10), chronic HBV-infected (CHB) patients (*n* = 12), and ACLF patients (*n* = 12) were stimulated with PMA (50 ng/mL) and ionomycin (1 µg/mL) in the presence of 10 µg/mL Brefeldin A for 5 h. Pam3Cys (1 µg/mL) was added or not as indicated. Cells were then intracellularly stained for IL-17A **(A)**, IL-22 **(B)**, TNF-α **(C)**, IL-21 **(D)**, and IFN-γ **(E)** and analyzed for the frequencies of cytokines positive CD4 T cells by flow cytometry. *P < 0.05, **P < 0.01, ***P < 0.001, compared to HS; ^#^*P* < 0.05, ^##^*P* < 0.01, ^###^*P* < 0.001, compared to CHB. Data are shown as the mean ± SD. Only differences with statistical significances were marked. Statistics analysis was performed by Mann–Whitney *U*-test and Wilcoxon matched pairs test.

Next, we investigated whether TLR2 signaling pathway activation could modulate Th17 cell response and/or function in ACLF patients. PBMCs isolated from HS, CHB patients and ACLF patients were stimulated with PMA and ionomycin for 5 h, and a TLR2 ligand Pam3Cys was added or not. As expected, CD4+ T cells of ACLF patients showed significantly increased Th17 effector cytokines (IL-17A, IL-22, TNF-α, and IL-21) production in response to PMA/ionomycin stimulation than that in HS and CHB patients. Importantly, Pam3Cys stimulation further enhanced all four cytokines production by CD4+ T cells of ACLF patients (Figures 7A–D). Pam3Cys stimulation also significantly enhanced IL-17A, IL-22, and TNF-α production by CD4+ T cells of CHB patients (Figures 7A–C). Interestingly, Pam3Cys stimulation could also enhance IFN-γ production by CD4+ T cells of both CHB and ACLF patients (Figure 7E). Taken together, our data indicated that TLR2 signaling pathway activation increased both Th17 and Th1 responses in HBV-infected patients.

## Discussion

Hepatitis B virus-related ACLF is a severe and life-threatening liver disease and previous studies have suggested the role of immune mediated injury in its pathophysiology (5). In the present study, we characterized the expression of TLRs in HBV-related ACLF patients and the role of TLR2 in disease aggravation. We observed a distinct TLRs expression pattern of PBMCs with increased TLR1/2/4/5/6/8/10 and decreased TLR3 levels in HBV-related ACLF patients. We could demonstrate that the TLR2 expression in peripheral CD4+ T cells was significantly increased in the ACLF patients compared to that in the HS and CHB patients, and was associated with Th17 responses as well as disease aggravation. Moreover, TLR2 ligand Pam3Cys stimulation promoted Th17 cell differentiation and response in the PBMCs of ACLF patients.

Toll-like receptors are a family of pattern-recognition receptors that triggers innate and adaptive immunity to pathogens and determines T cell differentiation and function (29, 32). It has been reported by us and others that TLRs signaling plays an important role in the eradication of invading HBV (33–36). Meanwhile, HBV manipulates TLR signaling pathways and altered TLRs expression was observed during HBV infection. However, the results of previous studies on alteration of TLR expression levels during HBV infection remain controversial. Chen et al. reported the expression of TLR2/4 mRNA was significantly lower in HBV infectious patients compared with healthy controls (37), while others showed that expression of TLR2/4 mRNA was increased in patients of CHB with active phase and CHB-related liver failure (22). Our result supports the latter report with the observation of increased TLR2/4 expression levels in PBMCs of CHB and even higher levels in HBV-related ALCF patients. TLR2 forms heterodimers with TLR1 or TLR6 to recognize lipopeptides and lipoteichoic acids of bacteria (38, 39). Strong upregulation of TLR1 and TLR6 expression was also observed in the ACLF patients in our study. The increased TLR1/2, TLR2/6, and TLR4 expression may lead to exaggerated immune responses triggered by bacterial infection in HBV-related ACLF patients, and thus promotes the deterioration of liver disease.

Upon activation by the innate immune system, naive CD4+ cells differentiate into distinct lineages of T helper cells depending on the environmental signals present. Among which, Th17 cells have been identified and characterized as a distinct T cell lineage mediating tissue inflammation, especially in autoimmunity (40). Previous studies have demonstrated Th17 cells might actively participate in liver injury of ACLF patients (15, 17, 41). A recent study demonstrated that IL-17 production by CD4+ T cells are strongly affected by TLR2, even after the early stages of lineage commitment (21). A direct stimulation with TLR2 agonists on CD4+ T cells promoted Th17 differentiation *in vitro* and led to more robust proliferation and Th17 cytokine production (21). This led us to analyzing the TLR2 expression on T cells and its correlation with Th17 response and disease aggravation in ACLF patients, which was the first clinical study on the topic to our knowledge. The results supported our hypothesis that the ACLF patients have increased TLR2 expression of T cells, which are positively correlated with Th17 responses and disease aggravation in the patients. Interestingly, higher TLR2 expression levels on T cells in HBeAg-negative patients than HBeAg-positive patients were observed in both the CHB and ACLF groups of our study. This is consistent with previous report that HBeAg may negatively regulate TLR2 expression in CHB patients (31). However, it remains unclear whether the increase of TLR2 expression is a consequence of HBeAg loss or it is a response of the immune system to against HBV which leads to HBeAg clearance in these patients. Notably, IFN-γ and TNF-α production were significantly increased in the HBV-ACLF patients, and both cytokines have been previously shown to mediate the upregulation of TLR2 expression during inflammation (42). Therefore, it is highly probably that the upregulation of TLR2 expression on T cells in HBV-ACLF patients is mediated by the increased production of inflammatory cytokines such as IFN-γ and TNF-α, especially during the course of HBeAg seroconversion. Moreover, it has been indicated that although increased expression of TLR2 was observed, its molecular signaling may be downregulated or inactivated by HBV (43). For instance, Wang et al. demonstrated that HBsAg suppresses TLR2/ligand-induced IL-12 production in monocytes/macrophages by blocking the JNK–MAPK pathway (44). Others reported that HBeAg inhibited TLR2-mediated activation of NF-κB and IFN-β (45). However, we could show that TLR2 ligand stimulation promoted the production of Th17 effector cytokines, such as IL-17a, IL-22, IL-21, and TNF-α, by CD4+ T cells from CHB and ACLF patients. This result indicated that the TLR2 signaling pathway for Th17 cell differentiation was maintained during HBV infection. Besides, increased production of inflammatory cytokines such as IL-6 and IL-10 was observed in the HBV-ACLF patients. IL-10 is usually regarded as an immunosuppressive cytokine, but was recently discovered to enhance acute liver immunopathology during HBV infection (46). In contrast, IL-6 is usually considered to be a major cytokine which promotes inflammatory responses during infection, but we recently discovered that TLR-induced IL-6 counter-regulates antiviral CD8+ T cell response (32). Thus, the upregulation of these cytokines may result in exacerbated liver injury and less controlled HBV replication in the HBV-ACLF patients. Further studies are needed to examine the detailed function of these cytokines in the pathophysiology of HBV-related ACLF.

Another question remains to be addressed is how TLR2 pathway is activated in these HBV-ACLF patients. It has been previously demonstrated that HBcAg can induce pro-inflammatory cytokine production by human THP-1 macrophages in a TLR2 dependent manner (47). Recently, Li et al. also showed that direct HBcAg stimulation induces TLR2 activation and IL-10 production of Kupffer cells (48). Therefore, viral proteins such as HBcAg may act as TLR2 agonists in HBV-ACLF patients. Besides, TLR2 is involved in the recognition of cell-wall components, lipoteichoic acid and lipoprotein of Gram-positive and Gram-negative bacteria. Bacterial translocations from the gut to portal circulation are very common during the early phase or the progressive phase of ACLF and may result in endotoxemia in the patients (49). Thus, increased bacteria components in the blood in HBV-ACLF patients may also trigger the activation of TLR2 signaling pathway in T cells.

Given the integral roles of TLRs in the initiation, propagation, and perpetuation of the inflammation in T cells, targeting TLRs has been considered as a preferred therapeutic strategy for the treatment of autoimmune disease (50). Antibodies targeting TLR2, OPN-305 and OPN-301, have been proved to be able to abrogate spontaneous cytokine release in rheumatoid arthritis (51). The results of our current study indicated that targeting TLR2 of T cells may represent an attractive therapeutic option for HBV-related ACLF and further studies are needed.

## Ethics Statement

This study was approved by the local medical ethics committee of Tongji Medical College, Huazhong University of Science and Technology with written informed consent from all subjects (2015LSZ-022). All subjects gave written informed consent in accordance with the Declaration of Helsinki.

## Author Contributions

CX, YL, XZ, XF, XY, JW, and BW performed the experiments; CX and JL analyzed the data; CX, JT, ML, DY, and JL wrote the manuscript. All authors read and approved the final manuscript.

## Conflict of Interest Statement

The authors declare that the research was conducted in the absence of any commercial or financial relationships that could be construed as a potential conflict of interest.

## Abbreviations

HBV, hepatitis B virus; ACLF, hepatitis B virus-associated acute-on-chronic liver failure; CHB, chronic hepatitis B; HS, healthy subjects; TLR2, toll-like receptor 2; Th17, T helper cell 17; PBMCs, peripheral blood mononuclear cells; PAMPs, pathogen-associated molecular patterns; EAE, experimental autoimmune encephalomyelitis; IL-6, interleukin-6; TGF-β, transforming growth factor-beta; TNF-α, tumor necrosis factor-α; TBIL, total bilirubin; DBIL, direct bilirubin; PT, prothrombin time; INR, international normalized ratio; WBC, white blood cell; ALB, serum albumin; PLT, platelet; PMA, Phorbol-12-myristate-13-acetate.
