# Corrigendum: TLR2 Expression in Peripheral CD4+ T Cells Promotes Th17 Response and Is Associated with Disease Aggravation of Hepatitis B Virus-Related Acute-On-Chronic Liver Failure

**DOI:** 10.3389/fimmu.2020.01566

**Published:** 2020-07-24

**Authors:** Chunli Xu, Yinping Lu, Xin Zheng, Xuemei Feng, Xuecheng Yang, Joerg Timm, Jun Wu, Baoju Wang, Mengji Lu, Dongliang Yang, Jia Liu

**Affiliations:** ^1^Department of Infectious Disease, Union Hospital, Tongji Medical College, Huazhong University of Science and Technology, Wuhan, China; ^2^Department of Anesthesiology, Union Hospital, Tongji Medical College, Huazhong University of Science and Technology, Wuhan, China; ^3^Institute for Virology, University Hospital, Heinrich-Heine-Universität Düsseldorf, Düsseldorf, Germany; ^4^Institute for Virology, University Hospital of Essen, University of Duisburg-Essen, Essen, Germany

**Keywords:** toll-like receptor 2, chronic hepatitis B, chronic hepatitis B-related liver failure, T helper cell 17, CD4+ T cells

In the original article, there was a mistake in Figure 2 as published. In panel B of the figure, the dot plot for TLR2+ CD8+ T cells was mistakenly placed where the dot plot for TLR2+ CD4+ T cells should have been placed, causing duplication in the figure. The corrected Figure 2 appears below.

**Figure 2:**
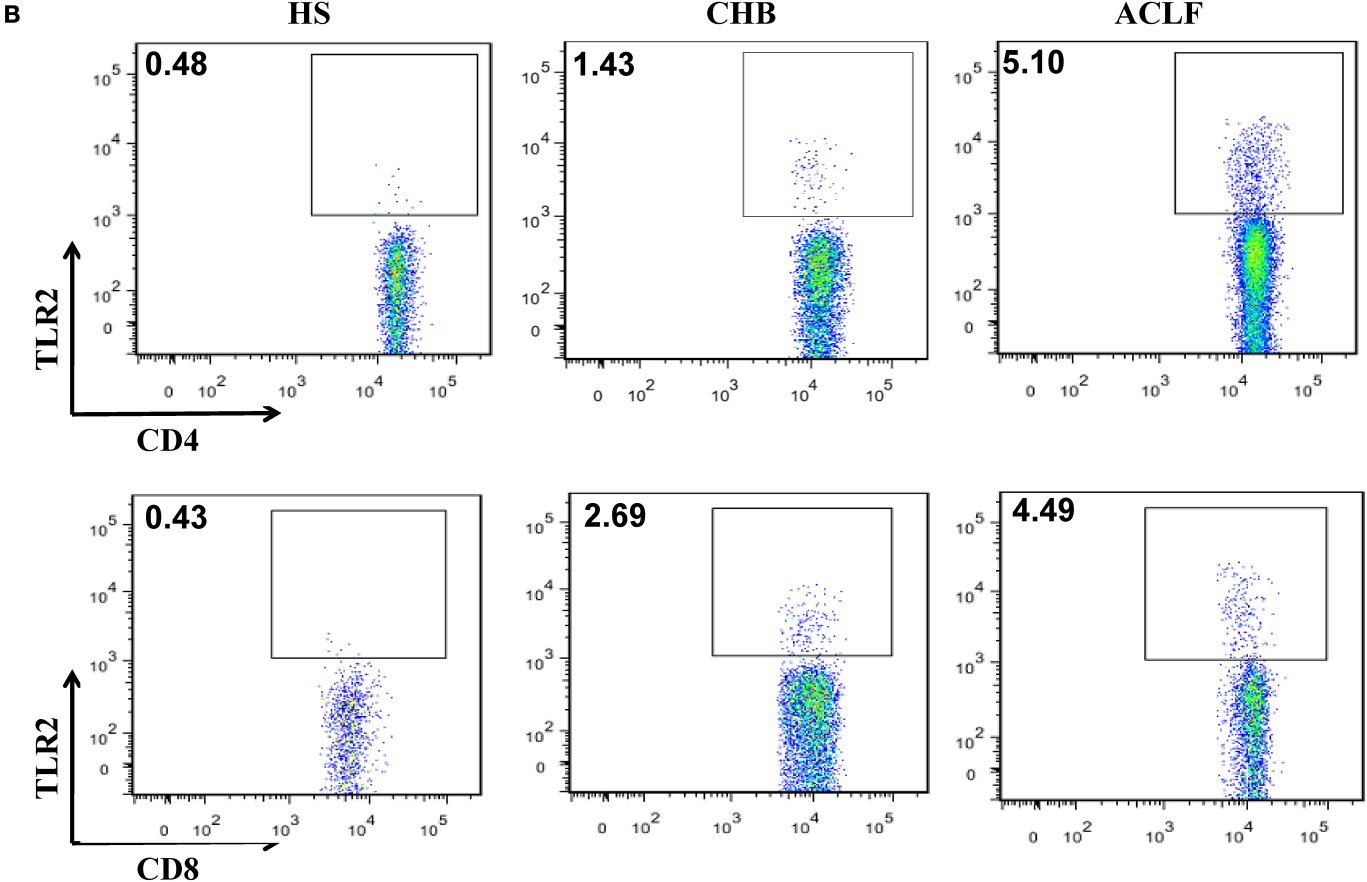


The authors apologize for this error and state that this does not change the scientific conclusions of the article in any way. The original article has been updated.

