# Supplementary material for: TLR2 Expression in Peripheral CD4+ T Cells Promotes Th17 Response and Is Associated with Disease Aggravation of Hepatitis B Virus-Related Acute-On-Chronic Liver Failure
Source: Front Immunol. 2017 Nov 23;8:1609. doi: 10.3389/fimmu.2017.01609 (PMC5703711; doi:10.3389/fimmu.2017.01609)
Supplement: Supplementary file 1 [file Data_Sheet_1.DOCX]

**TLR2 Expression in Peripheral CD4+T cells Promotes Th17 Response and Is Associated with Disease** **Aggravation of HBV-related Acute-on-chronic Liver Failure**

Chunli Xu^1,2^, Yinping Lu^1^, Xin Zheng^1^, Xuemei Feng^1^, Xuecheng Yang^1^, Joerg Timm^3^, Jun Wu^1^, Baoju Wang^1^, Mengji Lu^4^, Dongliang Yang^1,^*, Jia Liu^1,^*.

^1^Department of infectious Disease, Union Hospital, Tongji Medical College, Huazhong University of Science and Technology, Wuhan 430022, China.

^2^ Department of anesthesiology, Union Hospital, Tongji Medical College, Huazhong University of Science and Technology, Wuhan 430022, China.

^3^ Institute for Virology, Heinrich-Heine-University, University Hospital, Duesseldorf 40225, Germany.

^4^ Institute for Virology, University Hospital of Essen, University of Duisburg-Essen, Essen 45147, Germany

* These authors contributed equally to this work.

**Correspondence:**

Jia Liu

jialiu77@hotmail.com

Supplementary Table 1. Detailed clinical parameters of ACLF patients.

| Nr. | ALT | TB | INR | Ascites | HE | SBP | HRS | CF | Days between the acute decompensation initiation and the first sample collection |
| --- | --- | --- | --- | --- | --- | --- | --- | --- | --- |
| P1  P2 | 98  1432 | 277  298.6 | 1.9  2.05 | N  Y | Y  Y | N  N | N  N | N  N | 10  15 |
| P3 | 1175 | 319.9 | 1.87 | Y | N | Y | Y | N | 10 |
| P4 | 95 | 236.1 | 2 | Y | N | Y | N | N | 12 |
| P5 | 829 | 491 | 2.17 | Y | Y | Y | N | N | 10 |
| P6 | 905 | 218.5 | 2.59 | N | Y | N | y | Y | 15 |
| P7 | 1649 | 569.3 | 2.48 | Y | Y | Y | Y | Y | 5 |
| P8 | 452 | 467.9 | 2.34 | Y | Y | Y | Y | Y | 14 |
| P9 | 344 | 581 | 2 | Y | Y | Y | Y | N | 10 |
| P10 | 2156 | 350.9 | 2.01 | Y | N | N | N | N | 15 |
| P11  P12 | 614 | 368 | 2.12 | Y | N | Y | N | N | 15 |
|  | 1777 | 753 | 2.45 | N | Y | N | N | N | 10 |
| P13 | 97 | 451.8 | 1.9 | Y | N | Y | N | N | 10 |
| P14  P15 | 100 | 452.6 | 2.33 | Y | N | Y | N | N | 5 |
|  | 722 | 339.1 | 2.03 | Y | N | Y | N | N | 3 |
| P16 | 69 | 551.3 | 2.4 | Y | N | Y | N | N | 10 |
| P17 | 72 | 711.1 | 2.04 | Y | N | Y | Y | N | 15 |
| P18 | 103 | 859.7 | 2.31 | Y | Y | N | N | N | 3 |
| P19  P20 | 73  100 | 827  535.2 | 2.44  2.34 | Y  Y | Y  Y | Y  N | Y  N | N  N | 12  15 |
| P21 | 53 | 383.1 | 2.13 | Y | N | Y | Y | N | 15 |
| P22 | 91 | 527 | 2.54 | Y | N | N | N | N | 15 |
| P23 | 905 | 377 | 1.9 | N | Y | N | N | N | 15 |
| P24 | 99 | 249 | 2.57 | N | Y | N | N | N | 12 |
| P25 | 101 | 252.1 | 2.65 | Y | N | Y | N | N | 10 |
| P26 | 74 | 859 | 2 | Y | N | Y | N | N | 15 |

HE, hepatic encephalopathy. SBP, Spontaneous bacterial peritonitis. HRS，hepatorenal syndrome. CF, Circulation failure.

**A**

**B**

**Supplementary figure 1. TLR2 expression on CD14+ monocytes of PBMCs in the HS, CHB patients and ACLF patients.** The TLR2 expression on CD14+ monocytes of PBMCs in the HS, CHB patients and ACLF patients was determined by flow cytometry. The frequencies (A) and the mean fluorescence intensity (B) of TLR2 expression on CD14+ cells are shown. Statistics analysis was performed by Mann-Whitney U test.
